# The Complex Regulation of HIC (Human I-mfa Domain Containing Protein) Expression

**DOI:** 10.1371/journal.pone.0006152

**Published:** 2009-07-07

**Authors:** Ella Reiss-Sklan, Alexander Levitzki, Tally Naveh-Many

**Affiliations:** 1 Department of Biological Chemistry, Institute of Life Science, The Hebrew University, Jerusalem, Israel; 2 Minerva Center for Calcium and Bone Metabolism, Nephrology Services, Hadassah Hebrew University Medical Center, Jerusalem, Israel; Victor Chang Cardiac Research Institute (VCCRI), Australia

## Abstract

Human I-mfa domain containing protein (HIC) differentially regulates transcription from viral promoters. HIC affects the Wnt pathway, the JNK/SAPK pathway and the activity of positive transcription elongation factor-b (P-TEFb). Studies exploring HIC function in mammalian cells used ectopically expressed HIC due to undetected endogenous HIC protein. HIC mRNA contains exceptionally long 5′ and 3′ untranslated regions (UTRs) compared to the average length of mRNA UTRs. Here we show that HIC protein is subject to strict repression at multiple levels. The HIC mRNA UTRs reduce the expression of HIC or of a reporter protein: The HIC 3′-UTR decreases both HIC and reporter mRNA levels, whereas upstream open reading frames located in the 5′-UTR repress the translation of HIC or of the reporter protein. In addition, ectopically expressed HIC protein is degraded by the proteasome, with a half-life of approximately 1 h, suggesting that upon activation, HIC expression in cells may be transient. The strict regulation of HIC expression at the levels of mRNA stability, translation efficiency and protein stability suggests that expression of the HIC protein and its involvement in the various pathways is required only under specific cellular conditions.

## Introduction

The C-terminal region of HIC contains an 81 amino acid domain, which shares 77% identity and 81% similarity with the cysteine-rich C-terminal domain of the protein I-mfa. Hence, the name HIC for **H**uman **I**-mfa domain **C**ontaining protein. I-mfa (Inhibitor of MyoD Family A) inhibits the MyoD family of myogenic transcription factors [1], the Mash2 transcription factor involved in trophoblast differentiation, and TCF3 [2] and LEF1 [3], mediators of the Wnt pathway. Despite of the high homology between HIC and I-mfa, they appear to have different functions.

HIC was first identified as a protein that differentially regulates Tat-mediated and Tax-mediated expression of the human T-cell leukemia virus type I long terminal repeat (HTLV-I LTR) and the human immunodeficiency virus type I long terminal repeat (HIV-I LTR) [4]–[6]. HIC has also been reported to affect the Wnt pathway [3], the JNK/SAPK pathway [3] and the activity of positive transcription elongation factor-b (P-TEFb) [4], [7], [8]. Cigognini et al. recently studied chromosome 7 deletions in myeloid disorders [9]. 27% of acute myeloid leukemia (AML) and myelodysplastic syndrome (MSD) patients presented a chromosome 7 abnormality. The marker that showed the most frequent loss of heterozygosity is adjacent to HIC, hence, HIC has been proposed to be a candidate tumor suppressor gene. Although several studies have demonstrated that HIC is involved in a number of important signalling pathways and cellular processes, the exact role of HIC and the mechanism by which it affects the different pathways is still obscure. To date, studies exploring the role of HIC have been performed on over-expressed protein. No report of endogenous HIC protein has been published.

The mRNA encoding HIC contains a 590 nt 5′-untranslated region (UTR), a 741 nt coding sequence, and a 3276 nt 3′-UTR. Such UTRs are extremely long compared to the average length of UTRs of cellular mRNAs. The average length of the 5′-UTR in human mRNAs is 125–210 nt [10], [11] and the average length of the 3′-UTR is 1027 [10]. Long UTRs are usually involved in post-transcriptional regulation of mRNAs.

Post-transcriptional regulation of gene expression provides a key mechanism by which cells can rapidly change gene expression patterns in response to a variety of extracellular signals and disparate biological processes. mRNA-binding proteins interact with unique sequences in mRNAs to coordinately regulate their localization, translation and/or degradation. A common feature of many rapidly degraded mRNAs is the presence of AU-rich elements (AREs) in their 3′-UTRs. The sequence of this *cis*-element is variable, but frequently contains one or moreAUUUA pentameric motifs within or near a U-rich region [12]. Interactions between AREs and their specific binding proteins have diverse effects on target mRNAs. 5′-UTRs like 3′-UTRs, are deeply involved in post-transcriptional regulation of gene expression through specific mRNA motifs and RNA binding proteins. An increasing number of reports describe regulation of translation of specific mRNAs in response to specific stimuli. These mRNAs often contain a 5′-UTR considerably longer than the average cellular 5′-UTR [13], [14], may contain AUG codons upstream of the initiation codon for the main open reading frame, and have complex secondary structures [15].

Here we show that the expression of the HIC protein is subject to strict repression, reducing its expression to undetectable levels. We demonstrate that the HIC mRNA UTRs reduce the expression of HIC or of a reporter gene in transfected cells. The HIC 5′-UTR represses translation of HIC or of the reporter gene in a mechanism involving upstream open reading frames (uORFs), whereas the HIC 3′-UTR decreases the mRNA level. Ectopically expressed HIC protein is degraded by the proteasome with a half-life of approximately 1 h, suggesting that HIC protein expression in cells is transient even under conditions that elevate its translation in cells.

## Results

### Expression of HIC mRNA and Protein

**Figure 1:**
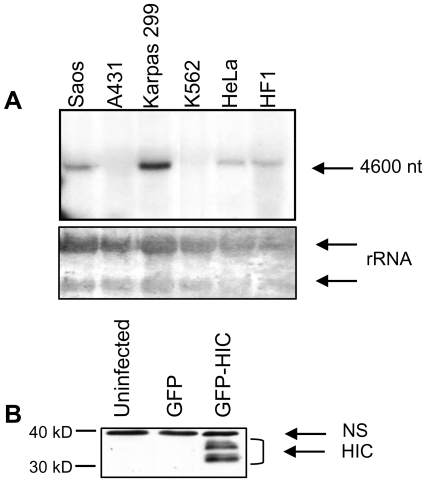
Expression of HIC mRNA and protein in different cell lines. A. Northern blot analysis of RNA from different cell lines, using a probe for HIC (upper panel). Lower panel- Methylene blue staining of the rRNA. B. Western blot analysis of uninfected HeLa cells or HeLa cells infected with viruses containing GFP (pAdeasy-CMV GFP) or GFP and the HIC coding sequence (pAdeasy-CMV GFP-HIC), expressed from two different sites in the pAdeasy bicystronic vector. Lysates were prepared 24 h after infection. HIC was detected using a serum generated against the N-terminus of the protein. 30 and 40 kD molecular weight markers are indicated.

HIC mRNA is expressed in the spleen, thymus, prostate, uterus, small intestine, peripheral blood leukocytes, but not in the testis and colon [5]. To compare expression of HIC mRNA in various human cell lines, we performed Northern blot analysis. A band of the expected size (∼4600 nt) was detected in Saos-2, Karpas 299, HeLa and HF1 cells, but not in A431 or K562 cells (Figure 1A). We could not detect endogenous HIC protein in any of these cell lines by Western blots using a rabbit polyclonal antibody that we generated against HIC (not shown and Fig. 1B, first lane). This antibody did however detect ectopic HIC over-expressed from a bicistronic vector encoding both GFP and the HIC ORF (Figure 1B). Western blots for samples expressing ectopic HIC revealed a 32-kDa doublet band of the expected molecular weight, and a higher molecular weight doublet band. The various bands may represent covalently modified protein (Figure 1B).

### HIC Untranslated Regions (UTRs) Inhibit the Expression of a Reporter Gene

**Figure 2:**
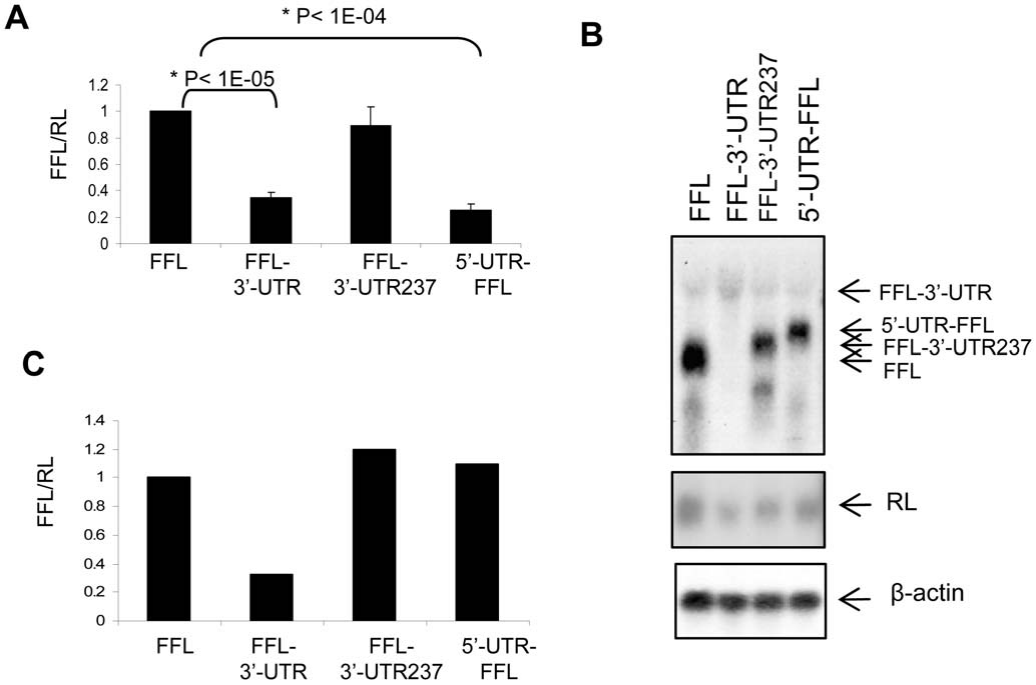
Expression of a FFL reporter gene is inhibited by the HIC UTRs. A. Luciferase assay of Saos cells transfected with constructs expressing a Firefly luciferase gene (FFL) or a FFL reporter gene fused to the HIC 3′-UTR (FFL-3′-UTR), the first 237 nucleotides of the HIC 3′-UTR (FFL-3′-UTR-237) or the 5′-UTR (5′-UTR-FFL). A Renilla luciferase (RL) encoding plasmid was added to each transfection mixture as a control for transfection efficiency. Cells were harvested 48 h post transfection and dual luciferase activities measured. FFL activity values were normalized to RL. The presented values are the average of three independent experiments preformed in duplicate. B. Northern blot analysis of RNA from Saos-2 cells transfected with the constructs described in A. RNA was extracted at 48 h and analyzed by Northern blots using probes for the FFL and RL genes and β-actin as a loading control. C. Quantification of the Northern blot shown in B. FFL mRNA level is normalized to RL mRNA level. Similar results were obtained in 2 more experiments.

We sought to examine whether the HIC mRNA UTRs affect the expression of a luciferase reporter protein in cells. We prepared constructs encoding the Firefly luciferase (FFL) reporter gene fused to the 5′-UTR of HIC (5′-UTR-FFL), the full length 3′-UTR (FFL-3′-UTR), or just 237 nt of the 5′ end of the 3′-UTR (FFL-3′-UTR-237). Saos-2 cells were transfected with each of the constructs. To control transfection efficiency all cells were co-transfected with a plasmid encoding Renilla luciferase (RL). 48 h after transfection the activities of FFL and RL were measured and FFL activity was normalized to RL activity. Fusion of the HIC 3′-UTR downstream to FFL reduced FFL activity by 65% (Figure 2A). Fusion of the first 237 nucleotides of the 3′-UTR did not have a significant effect on FFL expression. Fusion of the HIC 5′-UTR upstream to FFL reduced FFL activity by 75%. Similar results were obtained in all other cell lines examined (Figure S1).

To determine whether the decrease in FFL activity was due to reduced FFL mRNA or protein level, we purified RNA from cells expressing the UTR-FFL constructs and analyzed it on Northern blots using probes for FFL, RL and β-actin mRNAs. FFL mRNA levels were normalized to RL, as a measure of transfection efficiency and to β-actin, as a control for the amount of mRNA. mRNAs encoding FFL, RL and β-actin were detected in all samples (Figure 2B). Quantification of the intensities of the bands showed similar amounts of mRNA encoding FFL in cells transfected with constructs coding for the FFL gene, the FFL gene fused to the first 237 nucleotides of HIC 3′-UTR or the FFL gene fused to HIC 5′-UTR. In contrast, mRNA encoding FFL was barely detected in cells transfected with the FFL fused to the full length HIC mRNA 3′-UTR (Fig. 1B and C). Since all mRNAs were transcribed from the same CMV promoter, these results suggest that the low level of the 3′-UTR-expressing mRNA is caused by decreased mRNA stability. Both the 3′- and 5′-UTRs of HIC reduced FFL activity in cells (Fig. 2A). The fact that the HIC 3′-UTR but not the HIC 5′-UTR reduced FFL mRNA levels implies that the UTRs inhibit HIC protein expression by two different mechanisms. The HIC 3′-UTR causes a decrease in the mRNA level, most likely due to decreased mRNA stability, while the 5′-UTR inhibits mRNA translation.

### The 5′-UTR Represses HIC Translation in a Mechanism Involving Upstream Open Reading Frames (uORFs)

**Figure 3:**
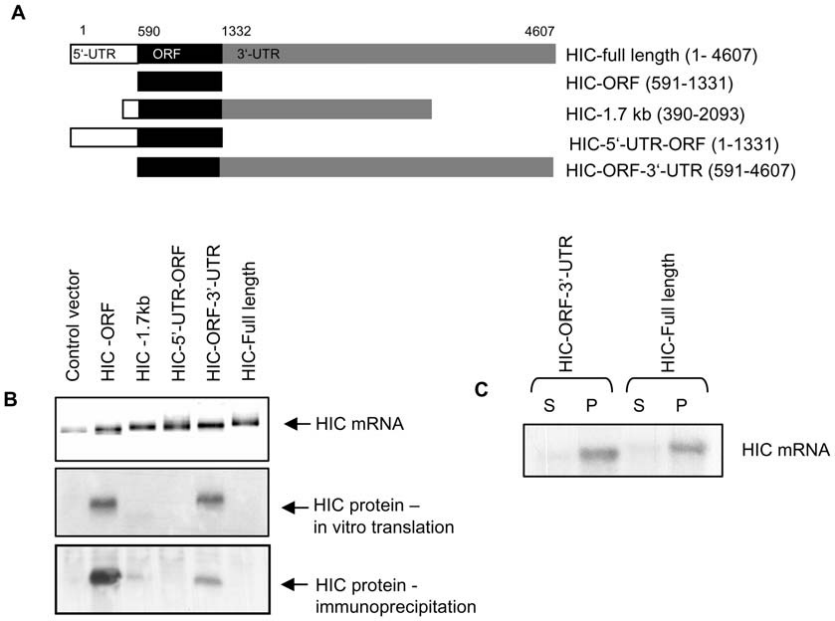
Translation of HIC is repressed by its 5′-UTR in vitro and in cells. A. Schematic representation of HIC fragments encoding the coding region or the coding region and various fragments of the HIC UTRs expressed from a pcDNA3 vector. B. Upper panel- Ethidium bromide stained gel of the RNAs transcribed *in vitro* using the different constructs in A. Middle panel- translation products from each RNA, translated *in vitro* in a reticulocyte lysate system in the presence of L-[35S]-methionine and L-[35S]-cysteine. Translation products were subjected to SDS-PAGE and visualized by autoradiography. Lower panel- Immunoprecipitation of HIC protein from HEK-293 cells transfected with the various HIC constructs. 24 h after transfection cells were metabolically labelled using L-[35S]-methionine and L-[35S]-cysteine for 11 hr. HIC was immunoprecipitated using anti-HIC serum, subjected to SDS-PAGE and visualized by autoradiography. C. Polysomal analysis of HEK-293 cells transfected with the HIC-ORF-3′UTR construct or a construct expressing the full length HIC mRNA. 24 h after transfection cytoplasmic extracts were prepared, fractionated by sucrose gradients and separated into polysomal (P) and sub-polysomal (S) fractions. RNA was analyzed by Northern blot using a probe for the HIC gene.

To determine whether the UTRs affect HIC expression in a manner similar to their effect on reporter FFL expression, we prepared a set of expression constructs encoding different parts of the HIC gene (Figure 3A). All constructs contained the HIC open reading frame (ORF). The HIC-FL construct contained the full length 4607 nt HIC cDNA. The HIC-ORF construct contained only the HIC coding region. The HIC-1.7 kb construct included the coding sequence as well as parts of the 5′ and 3′-UTRs. The HIC-5′-UTR-ORF construct consisted of the 5′-UTR and the coding region, and the HIC-ORF-3′-UTR construct included the coding region and 3′-UTR (Figure 3A).

We first examined HIC translation in an *in vitro* translation system. Equal amounts of in vitro transcribed RNA from the various constructs illustrated in figure 3A were translated using a rabbit reticulocyte lysate system (Fig. 3B). A product of the expected size was detected when transcripts containing the coding region only (HIC-ORF) or the coding region and the 3′UTR (HIC-ORF-3′-UTR) were translated *in vitro* (Figure 3B). In contrast, no product was detected from transcripts containing either the 3′ end of the 5′-UTR (HIC-1.7 kb) or the full length 5′-UTR (HIC-5′-UTR-ORF and HIC-full length). Consistent with the reporter gene assay (Figure 2), these findings indicate that translation of HIC mRNA is repressed by the 5′-UTR of HIC and that 200 nt at the 3′ end of the HIC mRNA 5′-UTR (in HIC-1.7 kb) are sufficient for translation inhibition of HIC *in vitro*.

We next examined the effects of the UTRs on HIC protein levels in cells. HEK-293 cells were transfected with expression plasmids encoding the various HIC constructs (illustrated in Figure 3A). To increase the sensitivity of HIC protein detection, cells were metabolically labeled using L-[^35^S]-methionine and L-[^35^S]-cysteine for 11 h. HIC was immunoprecipitated from the cell extracts, subjected to SDS-PAGE and visualized by autoradiography. HIC was immunoprecipitated from cells over-expressing the HIC-ORF (Figure 3B) and, to a lesser degree, from cells transfected with the HIC-ORF-3′-UTR construct. No HIC protein was detected in cells expressing the full length 5′-UTR (HIC-5′UTR-ORF and HIC-full length) or from cells expressing the 3′ end of the 5′-UTR (HIC-1.7 kb). Furthermore, no endogenous HIC was immunoprecipitated. This data is consistent with the *in vitro* translation experiment, confirming that the translation of HIC is repressed by the HIC mRNA 5′-UTR in transfected cells.

Although differing in their translation efficiency, mRNAs that included the HIC-5′-UTR as well as mRNAs that did not include the 5′-UTR were detected in the polysomal fraction in a polysomal profile analysis (Figure 3C). Therefore, the HIC mRNA is exported to the cytoplasm and not sequestered in the nucleus. Since the HIC protein is not translated in the cells, ribosomes may also be attached to the non-coding parts of HIC mRNA.

**Figure 4:**
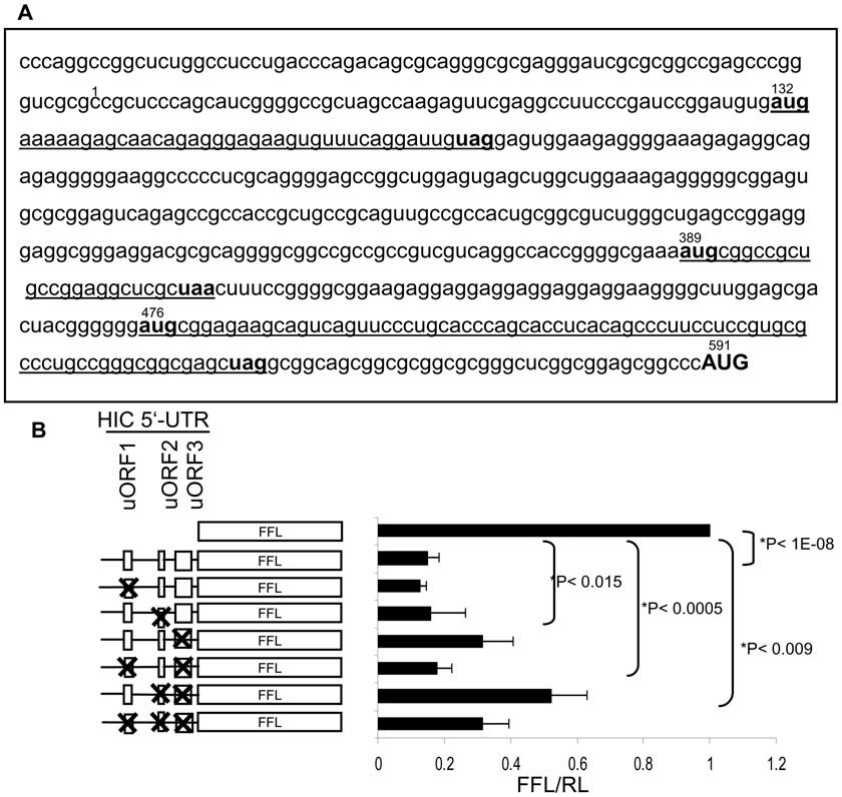
Mutations in the uORFs diminish the inhibitory effect of HIC 5′-UTR on FFL activity. A. Sequence of HIC 5′-UTR. Initiation and termination codons of the uORF are indicated in bold. uORFs are underlined. The HIC protein initiating codon is indicated by capital letters. Numbers denote the nucleotide number according to Genebank NM 199072 HIC cDNA sequence. B. A schematic representation of constructs expressing a FFL reporter gene, HIC-5′-UTR fused to the FFL gene, or HIC-5′-UTR-FFL in which one or more of the three uORF initiation codons were mutated to an AUC codon (left panel). Constructs were expressed in Saos2 cells together with a plasmid encoding Renilla luciferase as a control for transfection efficiency. Cells were harvested 24 h post transfection and dual luciferase activities measured. FFL activity values were normalized to RL. The presented values are the average of four independent experiments preformed in duplicate.

The sequence of the HIC mRNA 5′-UTR includes three short upstream open reading frames (uORFs) of 13, 8 and 25 amino acid lengths (Figure 4A). Short uORFs in the 5′-UTR affect translation efficiency of many eukaryotic genes [16]–[22]. To determine whether the potential uORFs in HIC 5′-UTR are involved in inhibition of its translation, we mutated the initiation codons (AUGs) of the uORFs, rendering them non-functional for initiation of translation in a set of constructs encoding the HIC 5′-UTR fused to a FFL reporter gene. The 5′UTR was cloned into an NcoI site in which the ATG start codon is included, thus all the nucleotides upstream to the ATG belong to the HIC sequence. In each construct, a single upstream AUG (uAUG) initiation codon or combinations of two or three uAUGs were mutated to AUC codons. Saos-2 cells were co-transfected with the various constructs and an RL expressing plasmid as a control for transfection efficiency. Dual luciferase activity was measured 24 h after transfection. Fusion of the HIC 5′-UTR upstream to FFL decreased its activity more than 5-fold (Figure 4B). A mutation in the initiation codon of uORF1 or uORF2 alone did not affect the activity of FFL. However, a mutation that abolished the initiation codon of uORF3 increased FFL activity 1.8-fold. Although neither uORF1 nor uORF2 elimination significantly affected the translation of the reporter gene when each was mutated separately, both mutations had an effect when mutated along with another uORF (uORF1 in combination with uORF3 or uORF2+3 and uORF2 in combination with uORF3 or uORF1+3). FFL activity was further increased (2.4-fold) by mutations in the initiation codons of both uORF2 and uORF3 suggesting an additive effect of the two mutations. Moreover, mutations in all three uORFs, increased FFL activity to a higher extant than the mutations in uORF1 and uORF3 only. Therefore, uORF2 may have a positive role in the inhibition of HIC translation. Surprisingly, mutations in the initiation codons for both uORF1 and uORF3 abolished the increase in FFL activity induced by mutation of uORF3 alone, suggesting that intact uORF1 leads to enhanced translation. Similarly, mutations in all three uORFs increased FFL activity to a lower extent than the mutations in uORF2 and uORF3 only. Therefore, uORF1 may have a positive role in the regulation of HIC translation. We conclude that translation of HIC is repressed by the 5′-UTR through uORF3 and uORF2. In contrast, uORF1 may have a positive role in translation of HIC mRNA.

### Stability of the HIC Protein

**Figure 5:**
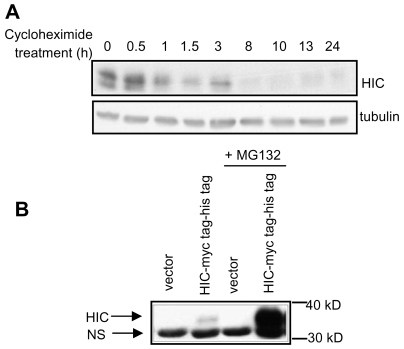
Ectopically expressed HIC protein has a short half life and is degraded by the proteosome. A. HEK-293 cells were transfected with a plasmid encoding HIC-myc tag (pcDNA3 HIC-myc tag-his tag). 24 h after transfection cells were treated with 100 µM cycloheximide for the indicated time periods and lysates analyzed by Western blots using anti-myc or anti-tubulin antibodies. B. HEK-293 cells were transfected with an empty vector or myc tagged HIC without or with addition of the proteosome inhibitor MG132 for 18 h. Lysates were analyzed by Western blot using an anti-myc antibody. 30 kD and 40 kD size markers are indicated. Arrows point to the HIC protein (HIC) and a non-specific band (NS).

To study HIC protein stability, we over-expressed HIC in HEK-293 cells using a construct encoding the ORF of HIC (without the UTRs) fused to myc and his tags. Expression of the HIC-myc tag-his tag following transfection of plasmid DNA was relatively low (Figure 5A) (compared to the expression using viral infection shown in figure 1). Transfected cells were treated with the translation inhibitor cycloheximide, harvested at various time points, and the level of the myc-tagged HIC protein was determined by Western blot. The half-life of exogenous HIC in HEK-293 cells was approximately 1 h (Figure 5A). Although the level of HIC was low when expressed from a plasmid vector, 18 h treatment with MG132, a proteasome inhibitor, significantly increased the amount of HIC detected in the cell extracts (Figure 5B). The elevation of HIC levels in cells treated with MG132 suggests that HIC is degraded by the proteasome. Therefore, expression of the HIC protein may be transient and limited by its short half life, as well as by the regulation of its translation and RNA stability.

## Discussion

Gene expression is a dynamic and tightly regulated process. A large number of transcription factors and other proteins regulating DNA methylation and chromatin structure are involved in transcriptional regulation. The transcribed mRNAs are regulated by multiple posttranscriptional mechanisms, including mRNA processing, editing, transport and stability [23]–[25]. Moreover, many mRNAs show lack of correlation between mRNA and protein levels [26]–[28], indicating that these genes are regulated at the level of translation. Changes in transcription rate as well as variations in mRNA decay rate, translation efficiency and protein stability play pivotal roles in regulation of protein expression.

HIC mRNA is expressed in several cell lines and tissues, but to date, no expression of endogenous HIC protein has been detected. Previous studies exploring the function of HIC in mammalian cells used ectopically expressed HIC. We have shown here that the expression of HIC is subject to complex regulation. The inability to detect HIC protein in these cells may be attributed to a large degree to repression of its expression. This includes repression at the levels of mRNA stability and of translation, mediated by HIC mRNA 3′- and 5′-UTRs. In addition, any HIC protein that is translated is rapidly degraded by the proteosome. We have demonstrated that the HIC 5′-UTR represses HIC translation, whereas the HIC 3′-UTR decreases its mRNA level (Figure 2, 3).

uORFs have been found to repress the translation of a number of mRNAs [16]–[22]. The presence of a few uORFs, or of an uORF which flanks or is proximal to the main ORF, greatly reduces the probability of reinitiation at the main ORF [29], [30]. We found that the third uORF plays the most significant role in the inhibition of translation by the HIC mRNA 5′-UTR (Figure 4), and that only when the initiation codon of this uORF was mutated, the effects of mutations in uORF1 and uORF2 could be detected. The second uORF was found to contribute to the inhibition of translation. In contrast, a mutation that abolished the first uORF decreased the effect of mutations in the following uORFs.

The finding that the first uORF has an opposite effect on translation efficiency implies that it may facilitate the reinitiation of translation in the downstream ORFs. A similar observation was reported for the translation of ATF4 (activating transcription factor 4), which is regulated by two uORFs with opposing roles [21], [31]. Lu et al. [21] and Vattem and Wek [31] suggested that uORF1 facilitates ribosome scanning and increases the reinitiation efficiency at a downstream ORF, uORF2, in unstressed cells or at the more distant main ORF in stressed cells.

The diverse impact of the different uORFs can be attributed to several factors, such as the distance between the main ORF and the uORF and the AUG context of the different ORFs. The efficiency of reinitiation improves as the spacing between two ORFs is lengthened [29], [30]. The distance between the termination codon of the third HIC uORF and the initiation codon of the HIC ORF is less than 40 nt. It is therefore likely that ribosomes initiating translation at the third uORF are precluded from translating HIC. The distances between the other ORFs are longer: 214 nt between uORF1 and uORF2 and 60 nt between uORF2 and uORF3 (Figure 4A). This is consistent with our finding that their inhibitory influence on translation of the main downstream ORF is minor.

A mutation in uORF1 or uORF2 alone did not affect the activity of FFL. This could be explained by the strong inhibitory effect of uORF3. Mutations in uORF1 or uORF2 may probably affect the translation efficiency of the following uORF, but not the translation of the main ORF. Translation of the main ORF would still be limited by uORF3, which is very proximate to the main ORF.

Although mutations in the initiation codons of HIC 5′-UTR enhanced translation of FFL, no combination of the mutations in the UORF was able to completely restore the activity of FFL. Thus, while the uORFs certainly have translational regulatory function, other factors found in the 5′-UTR of HIC may also repress HIC translation. Another uAUG followed by a stop codon at position 127 of the 5′-UTR may also contribute to the inhibition of HIC translation.

In mammals, the optimal consensus sequence for initiation is GCCA/GCC**AUG** G , with a purine at position −3 and a G at position +4 (underlined) being the most important nucleotides [32]. It is likely that the suboptimal context for the HIC translation initiation codon (CGGCCC**AUG**U) as well as the very long 5′ UTR containing other features (e.g. a complex RNA secondary structure) also contribute to the low translation efficiency induced by the 5′-UTR of HIC mRNA.

Fusion of the HIC 3′-UTR to a FFL reporter gene decreased FFL mRNA levels in the cells (Figure 2B). Since both the control FFL and the FFL fused to the 3′-UTR were expressed from a CMV promoter, we attribute the change in mRNA levels to altered mRNA stability. When over-expressed in cells, much more HIC protein was immunoprecipitated from cells transfected with a construct expressing HIC-ORF compared to the construct expressing HIC-ORF-3′-UTR (Figure 3C). Consistent with our results, Young et al. expressed HIC from a construct that contained HIC-ORF only or from a construct that contained the HIC-ORF and the 3′-UTR (both lacking the HIC 5′-UTR), and detected a much lower HIC protein level when the HIC construct included the 3′-UTR [33].

The half-life of an mRNA depends on sequences within the transcript itself, usually located in the 3′ UTR, and on RNA binding factors that interact with those sequences [34], [35]. Adenylate-uridylate rich elements (AREs) located in the 3′-UTR regulate the stability of various mRNAs encoding a wide repertoire of functionally diverse proteins [36]–[38]. AREs are found within U-rich regions and frequently contain one or more AUUUA motifs [36], [39]. The HIC 3′-UTR contains 18 repeats of the AUUUA consensus sequence, 16 of which are dispersed and two of which overlap. 35% of the HIC 3′-UTR is comprised of Uridylate nucleotides, some of which are clustered in long stretches (none of the clusters is included in the HIC-1.7 kb construct). Although the presence of AUUUA motifs in an AU-rich region does not guarantee the function of this motif in mRNA stability [40], it would be interesting to determine whether the numerous AUUUA repeats confer instability to HIC mRNA. However, HIC mRNA instability does not seem to be the major regulator of HIC repression. Endogenous HIC mRNA is expressed in various cell lines and tissues, but HIC protein is not detected in any of them. Therefore, expression of HIC protein expression is probably controlled primarily at the levels of translation and protein stability.

The level of ectopically expressed HIC protein was greatly increased by treatment with the proteasome inhibitor MG132, indicating that HIC is degraded by the proteasome (Figure 5A). The half-life of HIC is approximately 1 h (Figure 5B). Under conditions that stimulate translation, induction of endogenous HIC expression may still be transient due to the relatively short half-life of HIC. Thus, HIC expression is also regulated post-translationally. Under normal cellular conditions HIC is hardly translated and degradation by the proteasome may only act as a “backup” mechanism to prevent its expression. Nevertheless, under other yet unknown conditions in which HIC is translated, the regulation of HIC degradation may be more crucial and significant to the protein level.

HIC protein has been shown to interact with the cyclinT1 subunit of P-TEFb and with HIV-I Tat [4], [8], and inhibit Tat and p-TEFb dependent transcription from the HIV promoter [4]. In contrast, expression of HIC mRNA has been shown to activate transcription from the HIV-I promoter in a p-TEFb dependent manner [33]. Young et. al. sought to resolve this discrepancy and found that the 3′ end of HIC mRNA binds p-TEFb and activates it by displacing 7SK, an inhibitory small nuclear RNA (snRNA) [33]. Thus, HIC plays opposing roles in the regulation of P-TEFb: ectopically expressed HIC protein represses transcription from the HIV-I promoter while HIC mRNA elevates HIV-I promoter dependent translation. Activation of p-TEFb by HIC mRNA is presumably also required for general cellular transcription in the cell. We have evidence for a role for HIC in the stress response (submitted for publication), suggesting that HIC protein may be translated when the cell is subjected to certain forms of stress and/or as a protective mechanism against viral infection. Although the last 314 nt are sufficient for HIC mRNA to activate P-TEFb, an mRNA of 4607 nt is transcribed [33]. Inhibition of HIC translation during normal cell growth, by the long 5′UTR may also be required in order to keep HIC mRNA available and functional as an RNA molecule. At times of stress, reduced activation of HIC mRNA may be coordinated with induction of translation of the HIC protein.

We have shown here that the expression of ectopically expressed HIC protein is strongly repressed at the levels of mRNA stability, translation and protein stability. We suggest that endogenous HIC is down-regulated through similar mechanisms under normal cellular conditions. The tight suppression of HIC protein at multiple levels of gene expression may be withdrawn under certain, yet unidentified, cellular conditions. Further understanding of the complex regulation of HIC expression will contribute to the understanding of the cellular roles of HIC and the cellular pathways in which it is involved.

## Materials and Methods

Plasmids were prepared as detailed in Table 1.

**Table 1:** Plasmids.

Plasmid	Description
HIC-1.7 kb	Nucleotides 390–2093 of HIC were isolated from a human cDNA library constructed in vector pEBS7 [41], which was a kind gift from Prof. Legarski (The University of Texas, Huston, Texas, USA). The 1703 bp fragment was cloned into pcDNA3 (Invitrogen).
HIC-ORF	Nucleotides 591–1331 encoding the HIC open reading frame (ORF) were cloned into pcDNA3.
HIC-full length	The full length HIC cDNA clone was obtained from Prof. Mesnard (Institut de Biologie, Montpellier, France). Nucleotides 1–4607 were cloned in vector pcDNA3.
HIC-5′-ORF	Nucleotides 1–1331 encompassing HIC 5′-untranslated region (UTR) and the ORF were cloned into pcDNA3.
HIC-ORF-3′-UTR	Nucleotides 591–4607 including the HIC ORF and 3′-UTR were cloned into pcDNA3.
5′-UTR-FFL	The 5′-UTR fragment (nucleotides 1–590) of HIC was fused 5′ to a Firefly luciferase reporter gene (FFL) in the luciferase-expressing vector pGL3 (Promega). The 5′-UTR was inserted into an NcoI restriction site in which the luciferase AUG start codon is included.
FFL -3′-UTR237	Nucleotides 1332–1569 of HIC, including the beginning of the HIC 3′-UTR were fused 3′ to the Firefly luciferase reporter gene (FFL) in pGL3.
FFL -3′-UTR	The 3′-UTR fragment (nucleotides 1560–4607) of HIC was fused 3′ to a Firefly luciferase reporter gene (FFL) in the luciferase-expressing vector pGL3.
Mutated 5′-UTR-FFL	Point mutations were introduced into the construct 5′-UTR-FFL.In each construct one or more of the initiation codons in the uORFs of HIC 5′-UTR were mutated. The AUG initiation codons were replaced by AUC codons using the QuickChange Site Directed Mutagenesis kit (Stratagene, USA) according to the manufacturer's instructions.
pcDNA3-HIC-myc tag-his tag	A construct encoding the ORF of HIC fused to a myc tag and a his tag in the pcDNA3 vector was obtained from Prof. Mesnard (Institut de Biologie, Montpellier, France).

### Adenoviruses Expressing HIC

Adenoviruses expressing HIC were prepared as described in [42]. In brief, the HIC ORF was cloned into pAdTrack-CMV vector. pAdTrack-CMV- encoding HIC was digested with PmeI and inserted into pAdeasy using recombination in E.coli BJ5183.

### Cell Culture

All tissue culture reagents were purchased from Biological Industries Beit-Haemek LTD (Israel). MG132 was purchased from Calbiochem (USA). Cycloheximide was purchased from Sigma.

HEK-293, COS-7, HeLa, and A431 cells were grown in Dulbecco's modified eagle medium (DMEM) supplemented with 10% fetal calf serum (FCS). Osteosarcoma Saos-2 cells were grown in McCoy's medium supplemented with 10% FCS. Karpas299 and K562 cells were grown in RPMI medium supplemented with 10% FCS. HF1 cells were grown in keratinocyte growth medium (67% DMEM, 23% HAM/F12, 10% FBS, 5 µg/ml insulin, 2 nM T3, 5 µg/ml transferrin, 0.4 µg/ml hydrocortisone, 0.1 nM cholera toxin, 10 ng/ml EGF). All media were supplemented with 100 U/ml penicillin, 100 mg/ml streptomycin and all the cells were grown in a humid atmosphere containing 5% CO_2_ at 37°C.

### Cell Transfection and Infection

Cells were transfected using polyethyleneimine (PEI) as described previously [43]. Adenoviral infection was performed as described in [42].

### Northern Blot Analysis

1−2×10^6^ cells were seeded in 10 mm plates. 24–48 h after the plating, cells were transfected with the indicated plasmids. RNA was extracted from the cells using Tri-reagent (Sigma) or the RNeasy kit (Qiagen). 20 µg RNA were resolved on a 1% agarose gel containing 8% formaldehyde and capillary blotted onto a nylon membrane. The blot was then hybridized overnight at 42°C with ^32^P-labeled DNA probe, prepared with the Rediprime kit (GE Healthcare, USA) and exposed to film. For densitometry, sub-saturation exposures were analyzed using the NIH image 1.61 software.

### Immunoblotting

Equal amounts of protein in Laemmli sample buffer [44] were resolved by SDS-PAGE and electroblotted onto nitrocellulose membranes.

Antibodies: Anti-myc tag (9E10) was purchased from Santa Cruz Biotechnology Inc. (USA), Anti-tubulin was from Sigma. The anti-HIC serum was generated by immunization of rabbits with the peptide SGAGEALAPGPVG, comprising the first 13 amino acids of HIC.

### Immunoprecipitation of Radiolabeled HIC

10^6^ HEK-293 cells were seeded in 10 cm plates. The following day, cells were transfected with constructs encoding various parts of the HIC gene. 24 h after transfection, cells were labelled with 100 µCi/ml of Pro-mix (L-[^35^S]-methionine and L-[^35^S]-cysteine) (Amersham Pharmacia) for 11 h in DMEM lacking methionine and cysteine (Gibco) supplemented with 10% dialyzed FCS. Cells were washed 3 times with PBS and lysed in 0.1% SDS, 0.5% deoxycholate, 1% NP-40 in PBS supplemented with protease inhibitors (Sigma). Protein content was quantified using the micro Bradford assay [45].

HIC was immunoprecipitated from 1 mg protein lysate using anti-HIC epitope antibody (described above) coupled to protein G Sepharose (GE healthcare, USA). 25 µl of protein G were coupled to 20 µl anti-HIC serum by incubating the mixture for 2 h at 4°C in PBS containing 5% low-fat milk. Excess antibody was removed by washing the beads extensively with PBS. Lysates were incubated with coupled beads for 16 h at 4°C. Immunocomplexes were washed 4 times with lysis buffer. Then, 2×Laemmli sample buffer was added to the beads and the samples were boiled for 10 min. Immunocomplexes were resolved by SDS-PAGE and electroblotted. Nitrocellulose membranes were exposed to autoradiography.

### In Vitro Translation

RNAs were transcribed *in vitro* with T7 polymerase from linearized pcDNA3-HIC constructs using the Riboprobe *in vitro* Transcription System (Promega, USA). 0.5 µg of each purified transcript was translated using a rabbit reticulocyte lysate system (Promega, USA), according to the manufacturer's instructions, in the presence of 20 µCi Pro-mix (L- [^35^S]-methionine and L- [^35^S]-cysteine (Amersham Pharmacia Biotech) per reaction. Samples were resolved by SDS-PAGE, electroblotted onto nitrocellulose membranes, and membranes were exposed to autoradiography.

### Polysomal Fractionation

Polysomal fractionation using sucrose gradients was performed as described in [46].

### Reporter Assays

Cells were seeded in 12-well plates (6×10^4^/well). 48 h after seeding cells were transfected with the indicated plasmids. A plasmid encoding Renilla luciferase (pRL-PGK) was added to each transfection mixture as a control for transfection efficiency. 24–48 h after transfection cells were lysed with passive lysis buffer (Promega, USA). Firefly and Renilla luciferase activities were measured using a dual luciferase assay kit (Promega, USA).

## Supporting Information

Figure S1 Expression of a FFL reporter gene is inhibited by the HIC UTRs in various cell lines. Luciferase assay of various cell lines transfected with constructs expressing a Firefly luciferase gene (FFL) or a FFL reporter gene fused to the HIC 3′-UTR (FFL-3′UTR), the first 237 nucleotides of HIC 3′-UTR (FFL-3′UTR-237) or 5′-UTR (5′UTR-FFL). A Renilla luciferase (RL) encoding plasmid was added to each transfection mixture as a control for transfection efficiency. Cells were harvested 48 h post transfection and dual luciferase activities measured. FFL activity values were normalized to RL.
(0.46 MB TIF)
