# Supplementary figures and images for: The Complex Regulation of HIC (Human I-mfa Domain Containing Protein) Expression
Source: PLoS One. 2009 Jul 7;4(7):e6152. doi: 10.1371/journal.pone.0006152 (PMC2701633; doi:10.1371/journal.pone.0006152)

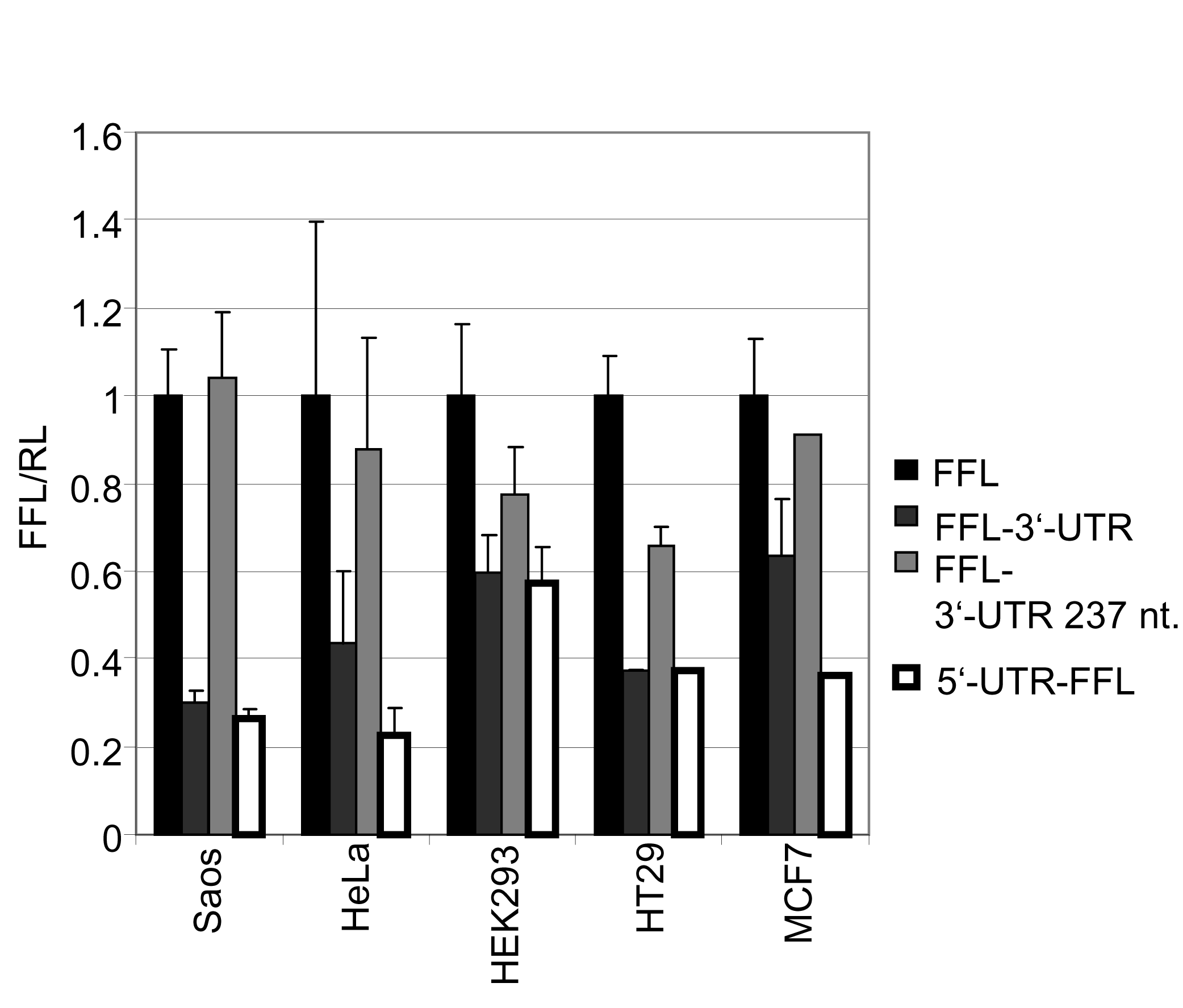

Supplement: Figure S1 — Expression of a FFL reporter gene is inhibited by the HIC UTRs in various cell lines. Luciferase assay of various cell lines transfected with constructs expressing a Firefly luciferase gene (FFL) or a FFL reporter gene fused to the HIC 3′-UTR (FFL-3′UTR), the first 237 nucleotides of HIC 3′-UTR (FFL-3′UTR-237) or 5′-UTR (5′UTR-FFL). A Renilla luciferase (RL) encoding plasmid was added to each transfection mixture as a control for transfection efficiency. Cells were harvested 48 h post transfection and dual luciferase activities measured. FFL activity values were normalized to RL. (0.46 MB TIF) [file pone.0006152.s001.tif]
